# DrSim: Similarity Learning for Transcriptional Phenotypic Drug Discovery

**DOI:** 10.1016/j.gpb.2022.09.006

**Published:** 2022-09-29

**Authors:** Zhiting Wei, Sheng Zhu, Xiaohan Chen, Chenyu Zhu, Bin Duan, Qi Liu

**Affiliations:** 1 Translational Medical Center for Stem Cell Therapy and Institute for Regenerative Medicine, Shanghai East Hospital, Bioinformatics Department, School of Life Sciences and Technology, Tongji University, Shanghai 200092, China; 2 Key Laboratory of Spine and Spinal Cord Injury Repair and Regeneration (Tongji University), Ministry of Education, Orthopaedic Department of Tongji Hospital, Bioinformatics Department, School of Life Sciences and Technology, Tongji University, Shanghai 200092, China; 3 Shanghai Research Institute for Intelligent Autonomous Systems, Shanghai 201210, China

**Keywords:** Metric learning, Transcriptional profile similarity, Drug annotation, Drug repositioning, LINCS

## Abstract

Transcriptional phenotypic drug discovery has achieved great success, and various compound perturbation-based data resources, such as connectivity map (CMap) and library of integrated network-based cellular signatures (**LINCS**), have been presented. Computational strategies fully mining these resources for phenotypic drug discovery have been proposed. Among them, the fundamental issue is to define the proper similarity between transcriptional profiles. Traditionally, such similarity has been defined in an unsupervised way. However, due to the high dimensionality and the existence of high noise in high-throughput data, similarity defined in the traditional way lacks robustness and has limited performance. To this end, we present DrSim, which is a learning-based framework that automatically infers similarity rather than defining it. We evaluated DrSim on publicly available *in vitro* and *in vivo* datasets in **drug annotation** and repositioning. The results indicated that DrSim outperforms the existing methods. In conclusion, by learning transcriptional similarity, DrSim facilitates the broad utility of high-throughput transcriptional perturbation data for phenotypic drug discovery. The source code and manual of DrSim are available at https://github.com/bm2-lab/DrSim/.

## Introduction

Compound perturbation-based transcriptional profiles in connectivity map (CMap) and library of integrated network-based cellular signatures (LINCS) have been successfully applied in drug discovery, such as elucidating the mechanisms of action (MOAs) for little-known compounds or suggesting new indications for existing drugs [1], [2], [3]. The process begins with a phenotype of interest to derive a query signature, *i.e.*, a set of differentially expressed genes. Then, the query signature is used to calculate similarities with compound perturbation profiles (also referred to as reference signatures) to indicate whether exposure to a specific compound is able to reverse or induce the phenotype of interest. In this process, the fundamental issue is to define the proper similarity between the query signature and the reference signature. Although various similarity methods, including Cosine [6], Kolmogorov-Smirnov statistic (KS) [4], Gene Set Enrichment analysis (GSEA) [5], [7], XSum [8], XCos [8], and statistically significant connections’ map (sscMap) [9], have been proposed to this end, a comprehensive study indicated that three issues remain. 1) All the existing methods are designed empirically in an unsupervised way. Due to the high dimensionality and the existence of high noise [10] in transcriptional signatures, it is difficult for empirically designed methods to characterize the similarity between transcriptional signatures, resulting in inherently limited performance. 2) Most of the existing similarity methods except for GSEA were developed specifically for CMap [7]. Despite the fashionability of CMap, its small scale restricts its application. Other resources, such as LINCS, which extended the CMap transcriptome data to a thousandfold scale-up, have been shown to be much more useful. However, data in these resources are heterogeneous. For example, data in LINCS are profiled by the L1000 array platform, which is different from the microarray platform used by CMap [4], [5]. This inconsistency may prevent existing empirically designed methods from achieving generalizable satisfactory performance. 3) The transcriptional profiles of cells responding to a compound perturbation are affected by the cell type and the duration of treatment [11]. However, none of the existing methods consider this characteristic in a detailed and appropriate way in performing similarity calculations between transcriptional signatures, which may lead to incorrect analysis results.

To this end, we present similarity learning for drug discovery (DrSim), which is a learning-based framework that automatically infers similarity rather than defining it. Basically, there are two main applications of such perturbation-based transcriptional profiles for phenotypic drug discovery: 1) drug annotation, *i.e.*, elucidating MOAs for less well-understood drugs, and 2) drug repositioning, *i.e.*, proposing new indications for existing drugs [1], [2], [3], [12]. Therefore, in our benchmark, we evaluated DrSim on publicly available *in vitro* and *in vivo* datasets in drug annotation and repositioning. Our comprehensive test results indicated that DrSim outperforms the existing methods. Taken together, by learning transcriptional similarity, DrSim facilitates the broad utility of high-throughput transcriptional perturbation data for phenotypic drug discovery with a conceptual improvement.

## Method

### The general framework of DrSim

**Figure 1:**
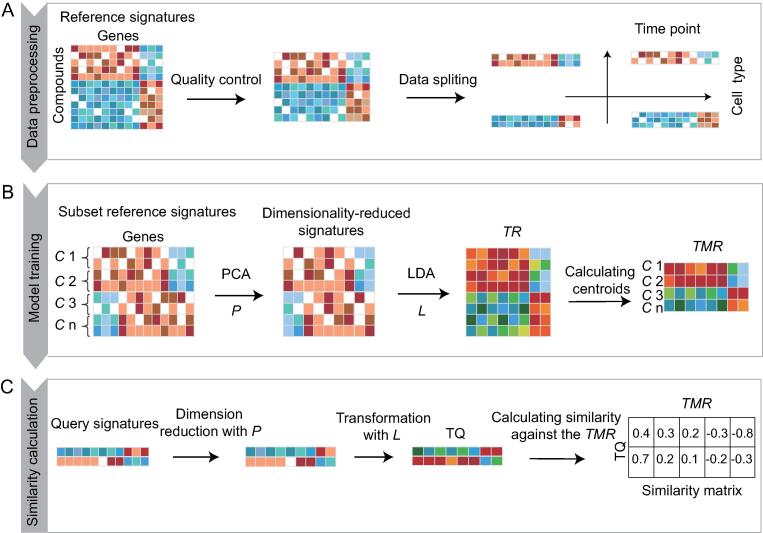
**The computational workflow of DrSim** DrSim comprises three main steps: data preprocessing, model training, and similarity calculation. **A.** In the first step, only signatures treated by compounds for 6 h or 24 h in the nine cancer cell lines and two non-cancer cell lines are retained, and the retained signatures are split into subsets according to the cell type and time point attributes. **B.** In the second step, DrSim automatically infers a similarity for query assignment based on the training reference signatures. First, PCA is applied to the reference signatures to reduce dimensionality. A transformation matrix *P* is obtained. Second, by applying LDA to the dimensionality-reduced signatures, a transformation matrix *L* is learned based on the signature labels indicating similarities and dissimilarities between them. The label of a signature is the compound that induces the signature. Finally, the *TRs* belonging to the identical compound are median centered to derive the *TMR*. The *TR* is calculated using Equation (1) defined in File S1. *C* denotes compound. **C.** In the third step, given a query signature, after transformation by *P* and *L*, its similarities to *TMRs* are calculated by cosine similarity [Equation (3) defined in File S1]. PCA, principal component analysis; LDA, linear discriminant analysis; *TR*, transformed reference; *TMR*, transformed median-centered reference; TQ, transformed query.

DrSim comprises three main steps: data preprocessing, model training, and similarity calculation (Figure 1).

### Data preprocessing

In our current study, for illustration purposes, LINCS is used as the data resource since it holds the largest-scale compound reference signatures [5], which presents millions of transcriptional profiles by treating various cancer cell lines with different compounds under different conditions. Nevertheless, the application of DrSim is not restricted to LINCS, and it can be applied directly to other compound perturbation-based datasets for phenotypic drug discovery. There are two steps in the data preprocessing stage. 1) Quality control. Only transcriptional signatures treated by compounds for 6 h or 24 h in the nine cancer cell lines (MCF7, A375, PC3, HT29, A549, BT20, VCAP, HCC515, and HEPG2) and two non-cancer cell lines (HA1E and NPC) in LINCS are retained, because most experiments were performed under these conditions. 2) Dataset splitting. The compound transcriptional signature contains four attributes (cell type, compound, time point, and dosage) since it was measured by treating a cell line with a compound under a certain concentration and at a particular time point. We evaluate the influence of these four attributes on the transcriptional signatures. As demonstrated in Figure S1A–D, the cell type, compound, and time point substantially influence the distribution of transcriptional signatures, while the compound dosage does not. Therefore, to minimize the influence of the cell line and time point in calculating the similarities between compound-induced signatures, 1) the signatures in LINCS are split into 22 subsets according to cell type and time point (Figure 1A; eleven kinds of cell lines and two kinds of time points); and 2) the subset signature that has identical cell type and time point attributes to the query is used as a reference when a query search is conducted. Since the compound dosage slightly impacts the distribution of transcriptional signatures, the signatures treated with identical compounds at different dosages are considered replicates.

### Model training

By adopting the linear discriminant analysis (LDA) metric learning algorithm (Figure S2A and B) [13], DrSim automatically infers a transcriptional similarity for query assignment based on the reference signatures. In the current study, the LDA algorithm was adopted since it achieves the best performance among all the metric learning algorithms (File S1) [13]. In summary, 1) principal component analysis (PCA) [14] is applied to the reference signatures to reduce dimensionality. A transformation matrix *P* is obtained. 2) By applying LDA to the dimensionality-reduced signatures, a transformation matrix *L* is learned based on the signature labels indicating similarities and dissimilarities between them. The label of a signature is the compound that induces the signature. The transformation matrix *L* that fits the relationships between signatures will project signatures into another space, in which signatures belonging to the identical class stay close to each other (intraclass similarity) while signatures belonging to different classes stay away from each other (interclass dissimilarity). In summary, the basic conception of LDA is to learn an optimal metric *L* that aims at maximizing intraclass similarity and interclass dissimilarity. 3) The transformed references (*TRs*) belonging to the identical compound are median centered to obtain the transformed median-centered reference (*TMR*; Figure 1C).

### Similarity calculation

For a query signature, after transformation by the *P* and *L* matrices, its similarities to *TMRs* are calculated by cosine similarity (File S1). The similarities are then ranked and can be used for query assignments. In drug annotation applications, the label of a query is assigned as the label of the reference that is most similar to the query since a positive score indicates compounds sharing a similar mechanism and activity [4], [5]. In drug repositioning applications, the compound that has the largest negative score for the query is suggested since a negative score indicates that exposure to a specific compound can reverse the phenotype of interest [4], [5].

### Evaluating the rationale of DrSim

**Figure 2:**
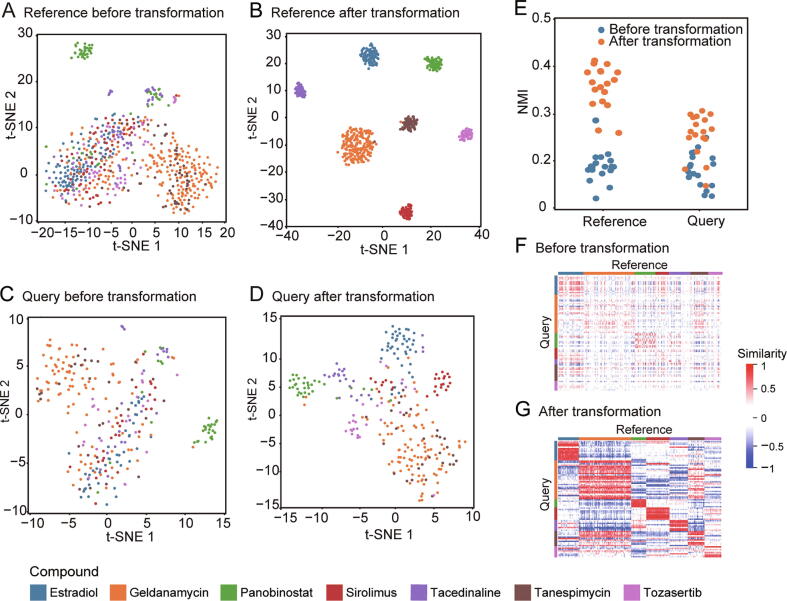
**The evaluation of the rationale of DrSim** **A.** Visualization of the clustering of reference signatures before transformation by the learned matrix *L*. **B.** Visualization of the clustering of reference signatures after transformation by the learned matrix *L*. Signatures belonging to the same class tend to cluster together, while signatures belonging to different classes tend to stay away from each other. **C.** Visualization of the clustering of query signatures before transformation by the learned matrix *L*. **D.** Visualization of the clustering of query signatures after transformation by the learned matrix *L*. Signatures belonging to the same class tend to cluster together, while signatures belonging to different classes tend to stay away from each other. **E.** After transformation by the learned matrix *L*, the NMIs of the reference and query signatures are significantly higher in the 22 subset datasets (*P* < 0.05). **F.** Heatmap of the similarities between the reference and query signatures before transformation by the learned matrix *L*. **G.** Heatmap of the similarities between the reference and query signatures after transformation by the learned matrix *L*. The query signatures and reference signatures belonging to the identical class become more similar, while those belonging to different classes become more dissimilar. NMI, normalized mutual information; t-SNE, t-distributed stochastic neighbor embedding.

Before evaluating the performance of DrSim, we demonstrated the rationale of the designed workflow to make sure that it is appropriate for drug annotation and drug repositioning based on transcriptional data. First, we evaluate whether the similarity learned by DrSim brings signatures with the same label close together, while separating signatures with different labels. More specifically, we employ t-distributed stochastic neighbor embedding (t-SNE) to visualize the distribution of query and reference signatures before and after applying DrSim. The parameter n_components of t-SNE is set to 2. Other parameters are set as default values. For illustration purposes, only signatures having the most replicates in MCF7 at 24 h are selected as an example. The selected signatures are split into queries and references at a ratio of 3:7, followed by applying DrSim. As shown in Figure 2A–D, after transformation by the learned matrix *L*, signatures coming from the same category have a tendency to cluster together, while signatures belonging to different classes tend to stay away from each other. Furthermore, to demonstrate that DrSim is still effective in a large-scale dataset, we employed the normalized mutual information (NMI) metric after applying DrSim to all the signatures having no less than 10 replicates in the 22 subset datasets [15] (File S1). If a clustering achieves a higher NMI, it has higher intraclass similarity and lower interclass similarity. As shown in Figure 2E, after applying DrSim, the NMIs of the reference and query signatures were significantly higher in the 22 subset datasets (*P* < 0.05).

Second, we visualized the distribution of similarities between reference signatures and query signatures before and after applying DrSim. As shown in Figure 2F and G, query signatures and reference signatures belonging to identical class become more similar, while query signatures and reference signatures belonging to different classes become more dissimilar. In other words, DrSim maximizes the similarity between signatures if they share a similar expression pattern. The characteristics of DrSim make it inherently suitable for drug annotation, since in drug annotation we assign a label to a query by searching for the most similar reference. Note that in drug repositioning, we intend to search for references that are most dissimilar to (maximally reverse) the query. To make the algorithm suitable for drug repositioning, we reversed the references before applying DrSim. In this case, DrSim maximizes the similarity between signatures if they have opposite expression patterns.

## Results

### Evaluating the performance of DrSim in drug annotation and drug repositioning

To demonstrate the advantage of DrSim, we compared it against six other commonly used methods, including Cosine, KS, GSEA, XSum, XCos, and sscMap. NFFinder [15] and L1000CDS [16] are not compared here because their core algorithms are KS and Cosine, respectively. The gene set size parameter, *i.e.*, the total number of bottom- and top-ranked differentially expressed genes in the six methods, was set to 200 as commonly used [17], [18]. Other parameters are set as default values. Note that DrSim employs PCA to reduce data dimensionality. Hence, to confirm that the performance improvement of DrSim benefits from using LDA rather than PCA, we also compare DrSim against a workflow that does not use LDA (referred to as no-LDA in our figures). In this study, DrSim was evaluated in two scenarios, *i.e.*, drug annotation and drug repositioning, based on publicly available *in vitro* and *in vivo* transcriptional dataset.

#### *Scenario 1*: *drug annotation*

**Figure 3:**
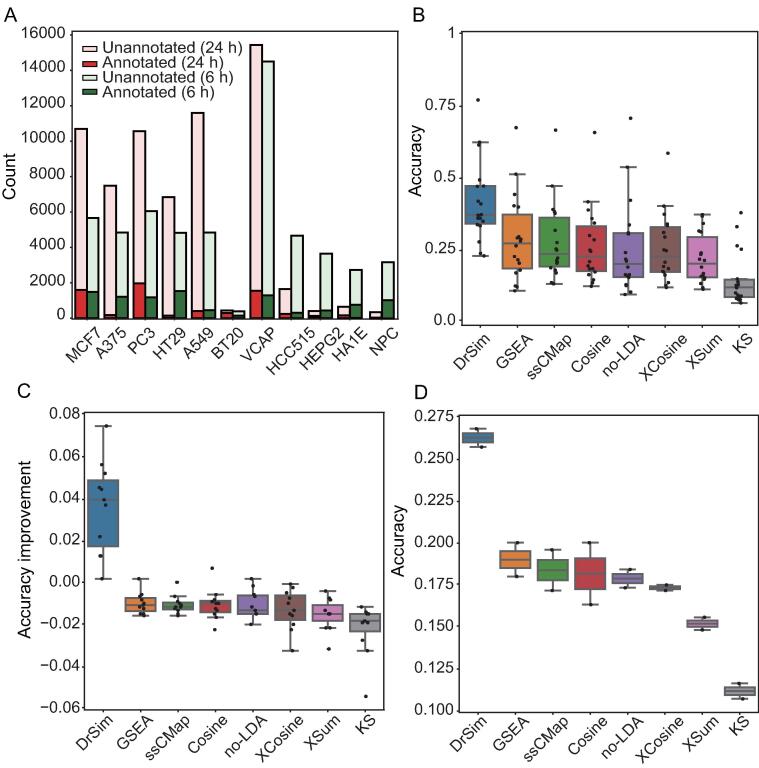
**Benchmark of DrSim against existing methods in drug annotation scenario** **A.** The proportion of compounds with MOA annotation information among all the compounds in CMap and LINCS. **B.** In predicting the MOAs of compounds, DrSim surpasses all the existing methods in all the subset data. **C.** As the training data size increases, only DrSim keeps the increase of accuracy. **D.** DrSim surpasses all the existing methods based on heterogeneous transcriptional profiles. MOA, mechanism of action; CMap, connectivity map; LINCS, library of integrated network-based cellular signatures.

The MOA describes how a drug produces its effect on the body. Compound perturbation-based transcriptional profiles have often been applied for drug annotation to uncover MOAs of less well-understood drugs. For example, the expression signature induced by thioridazine was found to have a strong similarity to those induced by DNA inhibitors, demonstrating that thioridazine exerts its anti-tumor activity by inhibiting DNA replication [19]. In the drug annotation benchmark scenario, the accuracy of predicting the MOAs of compounds was compared (see “calculation of the accuracy of predicting the MOAs of compounds” in File S1 for more details; Figure S3). The gold-standard MOAs of compounds in CMap and LINCS were retrieved from Huang and colleagues (Figure 3A; Table S1; File S1) [20]. As shown in Figure 3B, the accuracy of DrSim is higher and usually several times higher than that of the other methods. In addition, DrSim outperformed the no-LDA workflow, indicating that the performance improvement of DrSim benefits from employing the similarity learning-based strategy. In general, the training data size has an impact on the supervised learning model [21]. Therefore, we evaluated the impact of this factor on the accuracy of DrSim. Not unexpectedly, as the training data size increases, the accuracy of DrSim in predicting the MOAs of compounds increases (Figure 3C; File S1).

Compound-induced profiles are usually heterogeneous. For example, expression profiles in LINCS are measured by L1000 technology due to cost constraints [5], while most of the expression profiles used by researchers to derive a query are measured by RNA-seq and microarray technology [22]. To demonstrate that DrSim can achieve satisfactory performance in noisy and heterogeneous environments, heterogeneous signatures were used to predict the MOAs of compounds. Specifically, signatures in LINCS were used to predict the MOAs of compounds in CMap since the signatures in CMap and LINCS are profiled by microarray and L1000 technology, respectively. As illustrated in Figure 3D, although compared with that in the homogeneous scenario, the accuracies of all the methods drop in the heterogeneous scenario, DrSim still surpasses all the methods. This result demonstrates DrSim is tolerant of noisy data and heterogeneity (Figure 3B–D).

#### *Scenario 2*: *drug repositioning*

**Figure 4:**
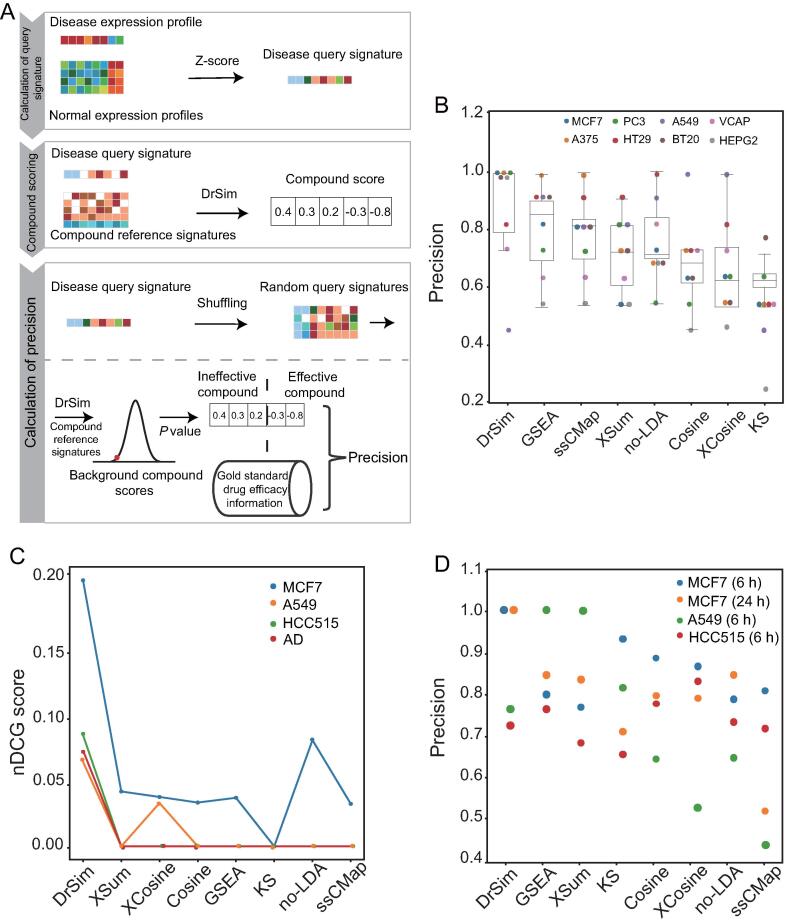
**Benchmark of DrSim against existing methods in drug repositioning scenario** **A.** In drug repositioning, the benchmark workflow comprises three main steps: calculation of disease query signature, compound scoring, and calculation of precision. 1) Disease query signature was generated by comparing disease expression profiles to normal expression profiles. Compound reference signatures were downloaded from LINCS. 2) Compounds were scored by computing the similarities between the compound reference signatures and the disease query signature with DrSim. 3) To determine whether a compound is effective against the input disease signature, its *P* value was computed by comparing its score with its background scores. Finally, by collecting gold standard drug efficacy information from public resources, the precision metric was calculated. **B.** DrSim surpasses all the existing methods in the *in vitro* datasets of eight cancer cell lines, indicating its high sensitivity. **C.** DrSim achieves the highest nDCG score in the BRCA, LUAD, and AD *in vivo* datasets, demonstrating its superiority. **D.** Of the BRCA and LUAD patients predicted by DrSim to respond to a drug, nearly all patients responded to it. For BRCA, MCF7 signatures were used as references; for LUAD, HCC515 and A549 signatures were used as references (File S1). BRCA, breast invasive carcinoma; LUAD, lung adenocarcinoma; AD, Alzheimer’s disease; nDCG, normalized discounted cumulative gain.

The suggestion of novel indications for existing drugs, *i.e.*, drug repositioning or repurposing, is significant for pharmaceutical study and is evolving as a method to reduce the cost of drug discovery [23]. Compound-induced transcriptional profiles have been extensively applied in this area, reflecting a paradigm shift in the pharmaceutical study, from the traditional seek of the magic bullet that targets a single pathogenic gene to the novel phenotypic means that inspect drug-gene-disease interactions from the system level. To comprehensively demonstrate the advantages of DrSim in transcription-based drug repositioning, we benchmarked it with existing methods in three areas, *i.e.*, *in vitro* datasets, *in vivo* datasets, and *in vivo* datasets with real-world evidence. We investigated whether DrSim can suggest effective compounds for query signatures that are derived from the phenotype of interest in the three datasets. The drug repositioning benchmark workflow comprises three steps: calculation of disease query signature, compound scoring, and calculation of precision (Figure 4A). More specifically, 1) disease query signature was generated by comparing disease expression profiles to normal expression profiles, and compound reference signatures were downloaded from LINCS. 2) Compounds were scored by computing the similarities between the compound reference signatures and the disease query signature with DrSim. 3) To determine whether a compound is effective against the input disease signature, its *P* value was computed by comparing its score with its background scores (see “calculation of the *P* value of a compound” in File S1 for more details). If the *P* value of a compound is less than 0.01, it was classified as effective, otherwise ineffective. Finally, by collecting gold standard drug efficacy information from public resources, the precision metric, *i.e.*, the proportion of real effective compounds among the predicted effective compounds, was calculated. Here, we focused on the precision metric rather than other metrics such as accuracy and recall because, in real applications, it is only practical to examine the few top indications. Therefore, we want the predicted effective compounds among the top suggestions to be truly effective as often as possible.

### Benchmarking DrSim in the *in vitro* datasets for drug repositioning

For the *in vitro* dataset, the performance of predicting effective compounds against cancer cell lines was compared. We analyzed eight kinds of cancer cell lines in view of the availability of compound-induced signatures and compound efficacy information on these cancer cell lines (Table S2). Following the benchmark workflow described above in the “drug repositioning” section, 1) query signatures were computed by comparing the expression profiles of the cancer cell line in the cancer cell line encyclopedia (CCLE) [24] to the expression profiles of corresponding normal tissue in genotype-tissue expression (GTEx; File S1) [25]. To minimize the time point attribute effect, the signatures profiled at 24 h in LINCS are used as the reference signatures since drug efficacy in genomics of drug sensitivity in cancer (GDSC), ChEMBL, and cancer therapeutics response portal (CTRP) is mostly measured at 24 h or 72 h. 2) Compounds were scored by computing the similarities between the compound reference signatures and the disease query signature with DrSim and other methods. 3) Compound was classified as effective or ineffective by comparing its score with its background scores (Figure 4A; File S1). Compound efficacy data were collected from GDSC, ChEMBL, and CTRP, which are the largest publicly available compound efficacy databases (File S1) [26], [27], [28]. As a result, DrSim obtained the highest precision (Figure 4B). Most of the effective compounds predicted by DrSim are truly effective.

### Benchmarking DrSim in the *in vivo* datasets for drug repositioning

For the *in vivo* dataset, the performance in predicting food and drug administration (FDA)-approved drugs against diseases was compared. Three kinds of cancer were analyzed, *i.e.*, lung adenocarcinoma (LUAD), breast invasive carcinoma (BRCA), and prostate adenocarcinoma (PRAD), based on the availability of FDA-approved drug reference signatures on these cancers (File S1). To further demonstrate the generalizability of DrSim, we apply it to a non-cancer disease, *i.e.*, Alzheimer’s disease (AD). It should be noted that AD patient-derived cell lines are not available in LINCS. Therefore, the signatures on the nine cancer cell lines in LINCS were used as references. Following the benchmark workflow described above in the “drug repositioning” section, 1) query signatures were collected by comparing cancer and AD patient expression profiles to the corresponding normal tissue expression profiles (File S1), and compound reference signatures in LINCS are used as the reference signatures; 2) compounds were scored by computing the similarities between the compound reference signatures and the disease query signature with DrSim and other methods; 3) compound was classified as effective or ineffective by comparing its score with its background scores (Figure 4A; File S1). As depicted in Table S3, only DrSim is able to properly identify FDA-approved drugs in BRCA, LUAD, and AD among all the tested methods, indicating its high sensitivity (Table S4). To quantitatively compare the ranking result, the normalized discounted cumulative gain (nDCG) was adopted (File S1). DrSim achieved the highest nDCG score in BRCA (MCF7), LUAD (HCC515 and A549), and AD (Figure 4C). Although none of the methods, including DrSim, predicted FDA-approved drugs in PRAD, several top-ranked effective drugs predicted by DrSim were validated *in vivo*. For example, calcitriol, the primary active metabolite of vitamin D, showed an anti-neoplastic effect in preclinical models of PRAD (Table S3) [29].

### Benchmarking DrSim in the *in vivo* datasets with real-world evidence for drug repositioning

For the *in vivo* dataset with real-world evidence, the performance in predicting drug response was compared. In view of the availability of drug response information in the cancer genome atlas (TCGA) and the reference signatures on those drugs, BRCA and LUAD patients were analyzed (File S1). Following the benchmark workflow described above in the “drug repositioning” section, 1) we collected 248 and 101 query signatures from BRCA and LUAD patients by comparing expression profiles from tumors to expression profiles from adjacent normal tissues. For BRCA patients, signatures at 6 h and 24 h in MCF7 were used as references. For LUAD patients, since two cell lineages of LUAD (A549 and HCC515) were profiled in LINCS, signatures in A549 and HCC515 were used as references. 2) Compounds were scored by computing the similarities between the compound reference signatures and the disease query signature with DrSim and other methods. 3) Compound was classified as effective or ineffective by comparing its score with its background scores (Figure 4A; File S1). If the *P* value of a drug was less than 0.01, we predicted that the patient would respond to the drug. The patients’ clinical outcomes were classified as “response” or “non-response” (File S1); as demonstrated in Figure 4D, DrSim outperformed the other methods. Of the patients predicted by DrSim to respond to a drug, nearly all the patients responded to it.

## Discussion

In the current study, we present DrSim, which is a learning-based framework for transcriptional phenotypic drug discovery. The similarity between signatures in DrSim is learned from data rather than being defined. Traditionally, such similarity has been defined in an unsupervised way, and due to the high dimensionality and the existence of high noise in these high-throughput data, it lacks robustness with limited performance. For example, in previous benchmark studies, XCos and XSum performed best in drug annotation on the CMap dataset [8], while GSEA and sscMap performed best on the LINCS dataset [17]. The robustness and superiority of DrSim on different platforms and data sources were demonstrated on *in vitro* and *in vivo* datasets. Taken together, DrSim facilitates the broad utility of high-throughput transcriptional perturbation data for phenotypic drug discovery.

The cell response to a perturbation is affected by the cell type as well as the duration of treatment (Figure S1A–D). However, none of the existing methods consider this characteristic in a detailed and appropriate way in performing similarity calculations between transcriptional signatures, which may lead to an incorrect analysis result. It is shown that the accuracy of predicting the MOAs of compounds drops if we do not consider this characteristic (Figure S4). This may be explained by the fact that although perturbations that show similar activity across cell types exist, the activities of most perturbations are cell type and time point specific. These perturbations usually target specialized proteins. For example, glucocorticoid receptor agonists are the maximum in cell lines in which the receptors of glucocorticoids are expressed [5]. In conclusion, cell type and time point attributes should be taken into consideration in calculating the similarity between transcriptional signatures.

The performance of DrSim improves when the training data size increases (Figure 3C). At present, nearly one-third of the perturbation-based expression profiles in CMap and LINCS have few replicates due to cost constraints [4], [5]. With the decreasing cost of high-throughput sequencing, perturbation-based expression profiles are accumulating rapidly. It is conceivable that the performance of DrSim can be further improved with such an increased amount of data.

Future improvements of DrSim include 1) designing a more efficient similarity-learning algorithm to characterize transcriptional similarity, and 2) identifying more efficient signatures through the genome-wide transcriptome. For example, using key pathway signatures to characterize the causality of diseases [30]. Then, such signatures could be incorporated into DrSim for phenotypic drug discovery.

## Code availability

Docker version of DrSim can be installed at https://hub.docker.com/r/bm2lab/drsim/. The usage and manual of DrSim are available at GitHub https://github.com/bm2-lab/DrSim/. The usage and manual of DrSim are also available at BioCode at National Genomics Data Center https://ngdc.cncb.ac.cn/biocode/tools/BT007273/.

## CRediT author statement

**Zhiting Wei:** Conceptualization, Data curation, Formal analysis, Investigation, Methodology, Writing - original draft, Validation, Writing - review & editing. **Sheng Zhu:** Data curation, Formal analysis, Methodology, Validation. **Xiaohan Chen:** Formal analysis, Visualization. **Chenyu Zhu:** Investigation, Visualization. **Bin Duan:** Investigation, Methodology. **Qi Liu:** Conceptualization, Writing - original draft, Validation, Writing - review & editing. All authors have read and approved the final manuscript.

## Competing interests

The authors declare that they have no competing interests.

## Supplementary material

Supplementary File S1 **Supplementary methods**

Supplementary Figure S1 **Visualization of the influence of the cell type, compound, time point, and compound dosage attributes on expression signature A.** Expression signatures profiled in the same cell line tend to cluster together, indicating that the cell type attribute impacts their distribution. **B.** Expression signatures profiled in the same compound tend to cluster together, indicating that the compound attribute impacts their distribution. **C.** Expression signatures profiled at the same time point tend to cluster together, indicating that the time point attribute impacts their distribution. **D.** Expression signatures profiled at different dosages tend to cluster together, indicating that the dosage attribute slightly impacts their distribution. t-SNE.

Supplementary Figure S2 **Benchmarking the metric learning algorithm A.** LDA is superior to NCA, LFDA, deepML, and MLKR in terms of accuracy in the drug annotation dataset. **B.** LDA is superior to NCA, LFDA, deepML, and MLKR in terms of runtime efficiency in the drug annotation dataset. LDA, linear discriminant analysis; NCA, neighborhood components analysis; LFDA, local fisher discriminant analysis; deepML, deep metric learning; MLKR, metric learning for kernel regression.

Supplementary Figure S3 **The strategy of splitting training and testing data in the drug annotation scenario** For a kind of MOA, half of the compounds were used as reference compounds and half were taken as query compounds. The signatures induced by the reference compounds were used for training and the signatures induced by the query compounds were used for testing. MOA, mechanism of action.

Supplementary Figure S4 **The accuracy of predicting the MOAs of compounds if we do not take the impact of cell type and time-point attributes into consideration** To evaluate the impact of cell type, signatures from different cell lines were used as queries and references, respectively. To evaluate the impact of time-point, signatures from different time points were used as queries and references, respectively. MOA, mechanism of action.

## References

[1] Qu X.A., Rajpal D.K. (2012). Applications of connectivity map in drug discovery and development. Drug Discov Today.

[2] Musa A., Ghoraie L.S., Zhang S.D., Glazko G., Yli-Harja O., Dehmer M. (2018). A review of connectivity map and computational approaches in pharmacogenomics. Brief Bioinform.

[3] Keenan A.B., Wojciechowicz M.L., Wang Z., Jagodnik K.M., Jenkins S.L., Lachmann A. (2019). Connectivity mapping: methods and applications. Annu Rev Biomed Data Sci.

[4] Lamb J., Crawford E.D., Peck D., Modell J.W., Blat I.C., Wrobel M.J. (2006). The connectivity map: using gene-expression signatures to connect small molecules, genes, and disease. Science.

[5] Subramanian A., Narayan R., Corsello S.M., Peck D.D., Natoli T.E., Lu X. (2017). A next generation connectivity map: L1000 platform and the first 1,000,000 profiles. Cell.

[6] Iorio F., Bosotti R., Scacheri E., Belcastro V., Mithbaokar P., Ferriero R. (2010). Discovery of drug mode of action and drug repositioning from transcriptional responses. Proc Natl Acad Sci U S A.

[7] Subramanian A., Tamayo P., Mootha V.K., Mukherjee S., Ebert B.L., Gillette M.A. (2005). Gene set enrichment analysis: a knowledge-based approach for interpreting genome-wide expression profiles. Proc Natl Acad Sci U S A.

[8] Cheng J., Yang L., Kumar V., Agarwal P. (2014). Systematic evaluation of connectivity map for disease indications. Genome Med.

[9] Zhang S.D., Gant T.W. (2008). A simple and robust method for connecting small-molecule drugs using gene-expression signatures. BMC Bioinformatics.

[10] Qiu Y., Lu T., Lim H., Xie L. (2020). A bayesian approach to accurate and robust signature detection on LINCS L1000 data. Bioinformatics.

[11] Niepel M., Hafner M., Duan Q., Wang Z., Paull E.O., Chung M. (2017). Common and cell-type specific responses to anti-cancer drugs revealed by high throughput transcript profiling. Nat Commun.

[12] Zheng Y., Peng H., Zhang X., Zhao Z., Gao X., Li J. (2019). Old drug repositioning and new drug discovery through similarity learning from drug-target joint feature spaces. BMC Bioinformatics.

[13] Izenman A.J., Izenman A.J. (2008). Modern Multivariate Statistical Techniques: Regression, Classification, and Manifold Learning.

[14] Wold S., Esbensen K., Geladi P. (1987). Principal component analysis. Chemometr Intell Lab.

[15] Setoain J., Franch M., Martínez M., Tabas-Madrid D., Sorzano C.O., Bakker A. (2015). NFFinder: an online bioinformatics tool for searching similar transcriptomics experiments in the context of drug repositioning. Nucleic Acids Res.

[16] Duan Q., Reid S.P., Clark N.R., Wang Z., Fernandez N.F., Rouillard A.D. (2016). L1000CDS2: LINCS L1000 characteristic direction signatures search engine. NPJ Syst Biol Appl.

[17] Lin K., Li L., Dai Y., Wang H., Teng S., Bao X. (2019). A comprehensive evaluation of connectivity methods for L1000 data. Brief Bioinform.

[18] Struckmann S., Ernst M., Fischer S., Mah N., Fuellen G., Möller S. (2021). Scoring functions for drug-effect similarity. Brief Bioinform.

[19] Rho S.B., Kim B.R., Kang S. (2011). A gene signature-based approach identifies thioridazine as an inhibitor of phosphatidylinositol-3′-kinase (PI3K)/AKT pathway in ovarian cancer cells. Gynecol Oncol.

[20] Huang C.T., Hsieh C.H., Chung Y.H., Oyang Y.J., Huang H.C., Juan H.F. (2019). Perturbational gene-expression signatures for combinatorial drug discovery. iScience.

[21] Ajiboye A.R., Abdullah-Arshah R., Qin H., Isah-Kebbe H. (2015). Evaluating the effect of dataset size on predictive model using supervised learning technique. Int J Comput Syst Softw Eng.

[22] Bansal V., Boucher C. (2019). Sequencing technologies and analyses: where have we been and where are we going?. iScience.

[23] Ashburn T.T., Thor K.B. (2004). Drug repositioning: identifying and developing new uses for existing drugs. Nat Rev Drug Discov.

[24] Barretina J., Caponigro G., Stransky N., Venkatesan K., Margolin A.A., Kim S. (2012). The cancer cell line encyclopedia enables predictive modelling of anticancer drug sensitivity. Nature.

[25] Lonsdale J., Thomas J., Salvatore M., Phillips R., Lo E., Shad S. (2013). The genotype-tissue expression (GTEx) project. Nat Genet.

[26] Gaulton A., Hersey A., Nowotka M., Bento A.P., Chambers J., Mendez D. (2016). The ChEMBL database in 2017. Nucleic Acids Res.

[27] Rees M.G., Seashore-Ludlow B., Cheah J.H., Adams D.J., Price E.V., Gill S. (2016). Correlating chemical sensitivity and basal gene expression reveals mechanism of action. Nat Chem Biol.

[28] Yang W., Soares J., Greninger P., Edelman E.J., Lightfoot H., Forbes S. (2012). Genomics of drug sensitivity in cancer (GDSC): a resource for therapeutic biomarker discovery in cancer cells. Nucleic Acids Res.

[29] Ben-Eltriki M., Deb S., Guns E.S.T. (2016). Calcitriol in combination therapy for prostate cancer: pharmacokinetic and pharmacodynamic interactions. J Cancer.

[30] Iwata M., Hirose L., Kohara H., Liao J., Sawada R., Akiyoshi S. (2018). Pathway-based drug repositioning for cancers: computational prediction and experimental validation. J Med Chem.

