# Supplementary material for: DrSim: Similarity Learning for Transcriptional Phenotypic Drug Discovery
Source: Genomics Proteomics Bioinformatics. 2022 Sep 29;20(5):1028–36. doi: 10.1016/j.gpb.2022.09.006 (PMC10025590; doi:10.1016/j.gpb.2022.09.006)
Supplement: Supplementary File S1 — Supplementary methods [file mmc1.docx]

**File S1 Supplementary methods**

**The general framework of DrSim**

*Data preprocessing*

The library of integrated network-based cellular signatures (LINCS) L1000 level-4 transcriptional signatures are calculated by comparing compound-induced profiles to control. All the associated metadata were downloaded from national center for biotechnology information sequence read archive (NCBI SRA) (GSE92742) and LINCS data portal 2.0 [1]. The raw signatures stored in GCTx format were converted to human-readable format by the cmapPy package [2]. To visualize the influence of the cell type, compound, time point, and dosage attributes on the distribution of compound-induced transcriptional signatures, we downloaded the processed transcriptional signatures from the L1000FWD website [3]. L1000FWD reduces the dimensionality of raw transcriptional signatures using principal component analysis (PCA), followed by performing t-distributed stochastic neighbor embedding (t-SNE) on them. For illustration purposes, downloaded processed signatures having the most replicates are selected as an example. As demonstrated in Figure S1A–D, the cell type, compound, and time point greatly impact the distribution of signatures, while the compound dosage does not.

*Model training and similarity calculation*

DrSim formulates a metric learning-based framework in which the similarity used for query assignment is learned from the reference data rather than being manually designed. Briefly, PCA was first applied to the reference data to reduce dimensionality. A transformation matrix *P* is obtained. Then, by applying linear discriminant analysis (LDA) [4] to the dimensionality-reduced signatures, a transformation matrix *L* is learned based on the signature labels indicating similarities and dissimilarities between signatures. The compound is used as the training label fed to LDA. In summary, the basic idea of LDA is to learn an optimal transformation matrix *L* that maximizes intraclass similarity and minimizes interclass dissimilarity. The transformed reference denoted as *TR* can be calculated as follows:

$$\begin{aligned} TR=R*P*L \#\left( 1 \right) \end{aligned}$$

Where *R* is the reference signature. The transformed references (*TRs*) belonging to the identical compound are median centered to obtain the transformed median-centered reference (denoted as *TMR*)*.* For a query signature Q, we transformed it as follows:

$$\begin{aligned} TQ=Q*P*L \#\left( 2 \right) \end{aligned}$$

Where *P* and *L* are obtained by PCA and LDA based on the reference signatures. Lastly, the similarity between the query signature and the *TMR* is computed by cosine similarity as follows:

$$\begin{aligned} similarity=\frac{TMR*TQ}{\left| \left| TMR \right| \right| \left| \left| TQ \right| \right|} \#\left( 3 \right) \end{aligned}$$

The n_components parameter in the PCA algorithm implemented in scikit-learn [5] was set to 0.98 (select the number of components so that the amount of explained variance in the reference signatures is greater than 98 percent). The n_components parameter in the LDA algorithm implemented in scikit-learn [5] was set to 50. Other parameters were set to default values.

*Benchmarking the metric learning algorithm*

To select the most appropriate metric learning algorithm, the performance of the commonly used metric learning algorithms, including LDA, neighborhood components analysis (NCA), local fisher discriminant analysis (LFDA), deep metric learning (deepML, <https://github.com/KevinMusgrave/pytorch-metric-learning>), and metric learning for kernel regression (MLKR) were compared in drug annotation dataset [6]. The drug annotation dataset rather than the drug repositioning dataset was used to benchmark their performances since the drug annotation gold standard was well defined. The large margin nearest neighbor (LMNN) algorithm was not considered since it takes too much time. The *n_components* parameter in LDA, NCA, LFDA, and MLKR is set to 50. Other parameters are set to default values. In deepML, the loss function and the optimizer are set to *TripletMarginMiner* and *Adam,* respectively. Other parameters are set to default values. As demonstrated in Figure S2A and B, LDA is superior to other methods in terms of accuracy and runtime efficiency. Therefore, LDA was adopted as the final metric learning algorithm in the current study.

*Calculation of the NMI*

In probability theory and information theory, normalized mutual information (NMI) [7] is used for measuring the amount of information of a clustering. To calculate the NMI of a collection of signatures, (1) a k-means clustering model was constructed to predict their clustering labels; (2) The NMI was calculated by comparing the predicted clustering labels with the true clustering labels of the given signatures. The k parameter in the k-means clustering model is set to the number of classes of the given signatures. Other parameters were set to default values.

**Test scenario 1: Drug annotation**

*Collection of compound MOA information*

The mechanisms of action (MOAs) of compounds in connectivity map (CMap) and LINCS were retrieved from Huang et al. [8]. The authors manually curated MOA tag descriptions for all compounds. In total, there are 213 kinds of MOA tag descriptions (Table S1). Among the 24,036 compounds in CMap and LINCS, only 2597 compounds have explicit MOA annotation.

*Calculation of the accuracy of predicting the MOAs of compounds*

The accuracy of predicting the MOAs of compounds was compared on the 22 subset datasets. For every subset dataset: (1) compounds inducing not less than five signatures and having explicit MOA annotation were kept. To make sure that a compound in the query is new to reference, the following strategy was adopted in splitting subset data into query and reference (Figure S3). For a kind of MOA, half of the compounds were used as reference compounds and half as query compounds. The signatures induced by the reference compounds were used for training and the signatures induced by the query compounds were used for testing. (2) For a query, its similarities to the references were calculated using DrSim, the no-LDA workflow, and the six other methods. The MOA of a query is then assigned as the MOA of the reference compound that is most similar to the query. (3) At last, we calculate the accuracy, *i.e.*, the proportion of correctly predicted queries among all the queries. To avoid uncertainty in splitting reference and query compounds, this procedure was repeated ten times and the average accuracy was used as the final result.

*Evaluation of the influence of training data size on DrSim*

To evaluate the impact of the training data size on the performance of DrSim and the six other methods, the following experiments were performed. We selected compounds inducing not less than 10 signatures in each subset of data. The signatures were split into query and reference as mentioned above. In the high replication scenario, all the signatures induced by a compound were used for training. In the low replication scenario, only half of the signatures induced by a compound were used for training. Finally, we compared the performances of DrSim between the high and low replication scenarios.

*Collection of query signatures from CMap for drug annotation*

CMap produces genome-wide transcriptional profiles from human cell lines treated with compounds at different dosages mainly for 6 h [9]. The current version (build 02) of CMap contains 7056 transcriptional profiles, including 6100 induced by treating cell lines with 1309 compounds and 956 induced by treating cell lines with dimethyl sulfoxide (DMSO) (control). CMap raw Affymetrix expression data and their annotations were downloaded from <https://portals.broadinstitute.org/cmap>. Only transcriptional profiles measured in MCF7 and PC3 (among the LINCS eleven cell lines) at 6 h (among the LINCS two time points) were kept. Expression data were fitted and normalized using the affyPLM [10] and affy [11] Bioconductor packages. The probe IDs in the microarray were converted to gene IDs using the hgu133a Bioconductor package. Expression levels of multiple probes matching the same gene were averaged as the expression level of that gene. Only genes measured both in CMap and LINCS platforms were retained. Finally, following the pipeline in LINCS, the robust z-scoring metric [12] was employed to derive query signatures as follows: the differential expression of gene $x$ in the $i$ sample within a batch were computed as follows:

$$\begin{aligned} z_{i}= \frac{x_{i}-median\left( X \right)}{1.4826*MAD \left( X \right)} \#\left( 4 \right) \end{aligned}$$

Where $X$ is the vector of normalized gene expression of gene *x* across all control samples within that batch, *MAD* is the median absolute deviation function, and the factor of 1.4826 makes the denominator a consistent estimator of scale for normally distributed data.

**Test scenario 2: Drug repositioning**

*Calculation of the p-value of a compound*

To determine whether a compound is effective against the input disease signature, its *P* value was computed by borrowing the idea from Subramanian et al. [12]. Briefly, (1) based on the LINCS compound reference signatures, a matrix *L* is learned. After transforming by the matrix *L*, the compound score *s* between a compound signature and the disease signature is calculated by cosine similarity (equation 3 in File S1); (2) We shuffled the disease signature 1000 times to generate a compendium of random disease signatures. For a specific compound, after transforming its signature and the random disease signatures by the matrix *L*, its background scores *S* were computed by equation 3 in File S1; (3) The *P* value of the compound against the disease is calculated as follows:

$$\begin{aligned} p= \frac{\sum_{i=1}^{1000} S_{i}\leq s}{1000} \#\left( 5 \right) \end{aligned}$$

*Calculation of query signatures in the in vitro dataset for drug repositioning*

The cancer cell line encyclopedia (CCLE) [13] contains gene transcriptional profiles from more than 1000 human cancer cell lines. We analyzed nine cancer cell lines among them, based on the availability of compound-induced signatures on these cancer cell lines in LINCS. The nine cancer cell line transcriptional profiles were downloaded from <https://portals.broadinstitute.org/ccle/>. Their corresponding normal tissue transcriptional profiles were downloaded from genotype-tissue expression (GTEx) [14], which collected transcriptional profiles from 54 non-diseased tissue sites across nearly 1000 individuals. For every cancer cell line, its transcriptional profile was merged with its corresponding normal tissue transcriptional profiles. The merged transcriptional profiles were then normalized using edgeR Bioconductor packages [15]. Genes not detected in LINCS L1000 technology were filtered. Following the pipeline in LINCS, the robust z-scoring metric was employed to derive the query signature for each cancer cell line [12] (calculated using equation 4 in File S1).

*Collection of drug efficacy information in the in vitro dataset for drug repositioning*

To be comprehensive, the gold standard drug efficacy information was downloaded from genomics of drug sensitivity in cancer (GDSC) [16], ChEMBL [17], and cancer therapeutics response portal (CTRP) [18]. GDSC and ChEMBL quantitatively measure drug efficacy against cancer cell lines using the half-maximal inhibitory concentration (IC50) metric while CTRP measures drug efficacy using the area under concentration-response (AUC) metric. In GDSC and ChEMBL, if the IC50 of a compound in a cell line is greater than 10000 nM, the compound is considered ineffective against the cell line, otherwise is considered effective [19]. In CTRP, if the AUC of a compound in a cell line is greater than the median AUC of all compounds in that cell line, the compound is considered ineffective, otherwise is considered as effective [18]. For a compound having multiple IC50s or AUCs in the three databases, we used the median to summarize them. Finally, compound efficacy information in GDSC, ChEMBL, and CTRP was merged. Compounds between LINCS and the three databases were mapped using the compound generic name. The compound efficacy information is available in the supplementary file (Table S2).

*Calculation of TCGA query signatures in the in vivo dataset for drug repositioning*

We collected RNA-seq transcriptional profiles of four kinds of cancers, including breast invasive carcinoma (BRCA), lung adenocarcinoma (LUAD), prostate adenocarcinoma (PRAD), skin cutaneous melanoma (SKCM) and their corresponding adjacent normal tissues from The Cancer Genome Atlas (TCGA) since only those cancers have food-and-drug administration (FDA) approved drug-induced reference signatures in LINCS. The SKCM was not for further analysis since there is not enough normal tissue transcriptional profile for the calculation of the query signature. Only genes measured in LINCS platforms were retained. Following the pipeline in LINCS, for every cancer type, the robust z-scoring metric was employed to derive the query signature (calculated using equation 3 in File S1). The gene Ensemble ID was converted to Entrez ID using clusterProfiler Bioconductor packages [20]. Genes not detected in the LINCS L1000 platform were filtered. Two cell lineages of LUAD (A549 and HCC515) and PRAD (PC3 and VCAP) are profiled in LINCS. Therefore, for LUAD, signatures at 6 h and 24 h in A549 and HCC515 were used as reference signatures; For PRAD, signatures at 6 h and 24 h in PC3 and VCAP were used as reference signatures; For BRCA, signatures at 6 h and 24 h in MCF7 were used as reference signatures. The predicted results at 6 h and 24 h were merged directly and sorted by the similarity score in Table S3.

*Calculation of Alzheimer query signatures in the in vivo dataset for drug repositioning*

The Alzheimer disease (AD) patient processed transcriptional profiles were downloaded from NCBI Gene Expression Omnibus (GEO, GSE26972). Samples from 3 female non-demented controls and 3 female AD patients were included in this study. Expression levels of multiple probes matching the same gene were averaged as the expression level of that gene. Only genes measured in LINCS platforms were retained. Following the pipeline in LINCS, the robust z-scoring metric was employed to derive the AD query signature (calculated using equation 3 in File S1). The gene Ensemble ID was converted to Entrez ID using clusterProfiler Bioconductor packages [20]. Genes not detected in the LINCS L1000 platform were filtered. For AD, the signatures on the nine cancer cell lines in LINCS were used as references since the AD patient-derived cell lines are not available. The predicted results at 6 h and 24 h were merged directly and sorted by the similarity score in Table S3.

*Collection of drug efficacy information in the in vivo dataset for drug repositioning*

In the *in vivo* benchmark scenario, the FDA approved drug information downloaded from National Institute of Health (NIH) was used as the ground truth for performance evaluation. Downloaded drugs were mapped to LINCS using the drug generic name. The FDA approved drug information used in this article is available in Table S4.

*Calculation of the query signatures in the in vivo data with real-world evidence for drug repositioning*

A part of the patients in TCGA received drug treatment. BRCA and LUAD patients were analyzed since only drugs treating BRCA and LUAD patients have reference signatures in LINCS. In total, 248 and 101 query signatures from BRCA and LUAD patients were available by comparing transcriptional profiles from tumors to those from adjacent normal tissues. For BRCA patients, signatures at 6 h and 24 h in MCF7 were used as references. For LUAD patients, since two cell lineages of LUAD (A549 and HCC515) were profiled in LINCS, signatures at 6 h in A549 and HCC515 were used as references (signatures were not available at 24 h in A549 and HCC515 for those drugs in TCGA records).

*Collection of the drug efficacy information in the in vivo data with real-world evidence for drug repositioning*

The drug response records of BRCA and LUAD patients in TCGA were used as the ground truth to evaluate the performance of predicting drug response. The “complete response” and “partial response” in the records were classified as “response” and the “clinical progressive disease” and “stable disease” in the records were classified as “non response”.

*Calculation of the normalized discounted cumulative gain*

Normalized discounted cumulative gain (nDCG) is a measure of the ranking quality. In the current study, nDCG measures the gain of an FDA approved drug based on its rank position in the result list. The gain is accumulated from the top of the result list to the bottom, with the gain of each result discounted at lower ranks. nDCG is calculated as follows:

$$\begin{aligned} nDCG= \frac{\sum_{i=1}^{j} \frac{1}{{log}_{2}\left( R\left( i \right)+1 \right)}}{\sum_{i=1}^{N} \frac{1}{{log}_{2}\left( R\left( i \right)+1 \right)}} \#\left( 6 \right) \end{aligned}$$

Where $j$ is the number of FDA approved drugs predicted by DrSim and the other commonly used methods, $N$ is the number of predicted effective drugs, $R(i)$ is the rank of a predicted drug.

**References**

[1] Stathias V, Turner J, Koleti A, Vidovic D, Cooper D, Fazel-Najafabadi M, et al. LINCS data portal 2.0: next generation access point for perturbation-response signatures. Nucleic Acids Res 2020;48:D431–9.

[2] Enache OM, Lahr DL, Natoli TE, Litichevskiy L, Wadden D, Flynn C, et al. The GCTx format and cmap{Py, R, M, J} packages: resources for optimized storage and integrated traversal of annotated dense matrices. Bioinformatics 2018;35:1427–9.

[3] Wang Z, Lachmann A, Keenan AB, Ma'ayan A. L1000FWD: fireworks visualization of drug-induced transcriptomic signatures. Bioinformatics 2018;34:2150–2.

[4] Izenman AJ. Linear discriminant analysis. In: Izenman A. J. (ed) Modern Multivariate Statistical Techniques: Regression, Classification, and Manifold Learning. New York, NY: Springer New York, 2008, 237–80.

[5] Pedregosa F, Varoquaux G, Gramfort A, Michel V, Thirion B, Grisel O, et al. Scikit-learn: machine learning in python. J Mach Learn Res 2011;12:2825–30.

[6] Vazelhes Wd, Carey C, Tang Y, Vauquier N, Bellet A. Metric-learn: metric learning algorithms in python. J Mach Learn Res 2020;21:1–6.

[7] Estevez PA, Tesmer M, Perez CA, Zurada JM. Normalized mutual information feature selection. IEEE Transactions on Neural Networks 2009;20:189–201.

[8] Huang CT, Hsieh CH, Chung YH, Oyang YJ, Huang HC, Juan HF. Perturbational gene-expression signatures for combinatorial drug discovery. iScience 2019;15:291–306.

[9] Lamb J, Crawford ED, Peck D, Modell JW, Blat IC, Wrobel MJ, et al. The connectivity map: using gene-expression signatures to connect small molecules, genes, and disease. Science 2006;313:1929–35.

[10] Brettschneider J, Collin F, Bolstad BM, Speed TP. Quality assessment for short oligonucleotide microarray data. Technometrics 2008;50:241–64.

[11] Gautier L, Cope L, Bolstad BM, Irizarry RA. Affy—analysis of affymetrix genechip data at the probe level. Bioinformatics 2004;20:307–15.

[12] Subramanian A, Narayan R, Corsello SM, Peck DD, Natoli TE, Lu X, et al. A next generation connectivity map: L1000 platform and the first 1,000,000 profiles. Cell 2017;171:1437–52.e17.

[13] Barretina J, Caponigro G, Stransky N, Venkatesan K, Margolin AA, Kim S, et al. The cancer cell line encyclopedia enables predictive modelling of anticancer drug sensitivity. Nature 2012;483:603–7.

[14] Lonsdale J, Thomas J, Salvatore M, Phillips R, Lo E, Shad S, et al. The genotype-tissue expression (GTEx) project. Nat Genetics 2013;45:580–5.

[15] Robinson MD, McCarthy DJ, Smyth GK. EdgeR: a bioconductor package for differential expression analysis of digital gene expression data. Bioinformatics 2009;26:139–40.

[16] Yang W, Soares J, Greninger P, Edelman EJ, Lightfoot H, Forbes S, et al. Genomics of drug sensitivity in cancer (GDSC): a resource for therapeutic biomarker discovery in cancer cells. Nucleic Acids Res 2012;41:D955–61.

[17] Gaulton A, Hersey A, Nowotka M, Bento AP, Chambers J, Mendez D, et al. The ChEMBL database in 2017. Nucleic Acids Res 2016;45:D945–54.

[18] Rees MG, Seashore-Ludlow B, Cheah JH, Adams DJ, Price EV, Gill S, et al. Correlating chemical sensitivity and basal gene expression reveals mechanism of action. Nat Chem Biol 2016;12:109–16.

[19] Chen B, Ma L, Paik H, Sirota M, Wei W, Chua MS, et al. Reversal of cancer gene expression correlates with drug efficacy and reveals therapeutic targets. Nat Commun 2017;8:16022.

[20] Yu G, Wang LG, Han Y, He QY. ClusterProfiler: an r package for comparing biological themes among gene clusters. OMICS 2012;16:284–7.
