# Supplementary figures and images for: DrSim: Similarity Learning for Transcriptional Phenotypic Drug Discovery
Source: Genomics Proteomics Bioinformatics. 2022 Sep 29;20(5):1028–36. doi: 10.1016/j.gpb.2022.09.006 (PMC10025590; doi:10.1016/j.gpb.2022.09.006)

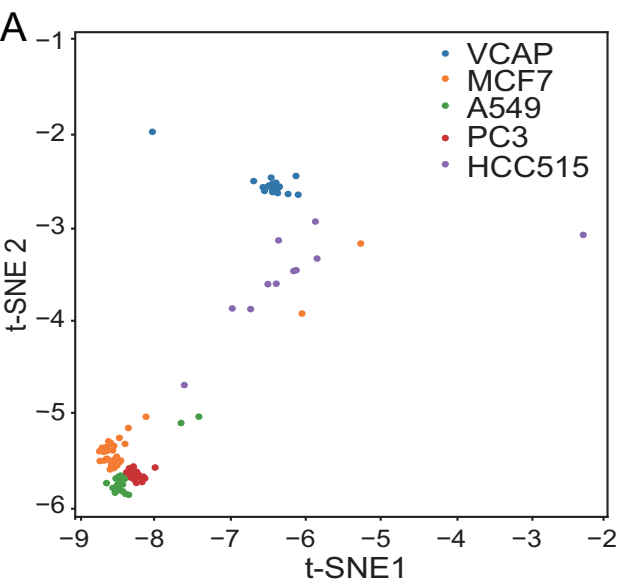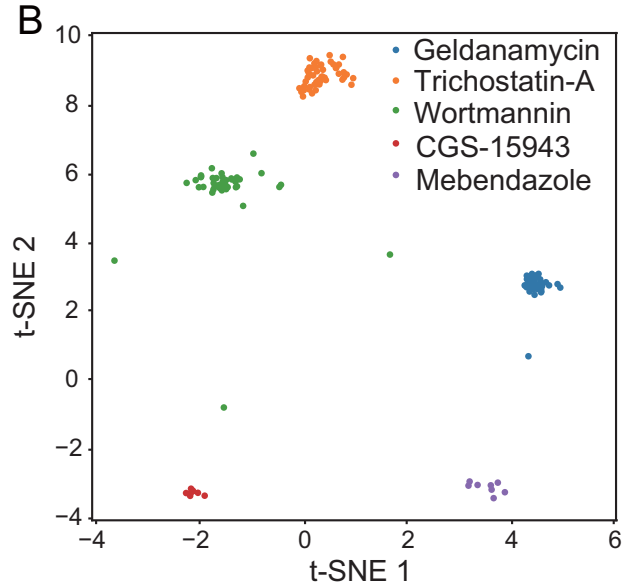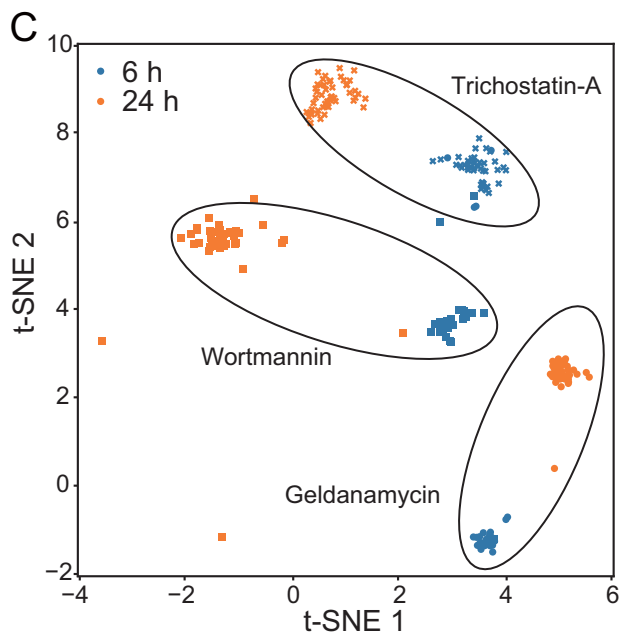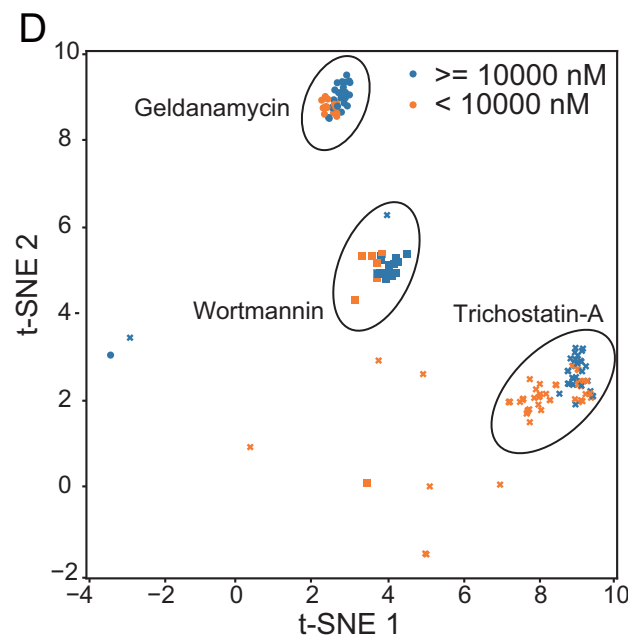

Supplement: Supplementary Figure S1 — Visualization of the influence of the cell type, compound, time point, and compound dosage attributes on expression signature A. Expression signatures profiled in the same cell line tend to cluster together, indicating that the cell type attribute impacts their distribution. B. Expression signatures profiled in the same compound tend to cluster together, indicating that the compound attribute impacts their distribution. C. Expression signatures profiled at the same time point tend to cluster together, indicating that the time point attribute impacts their distribution. D. Expression signatures profiled at different dosages tend to cluster together, indicating that the dosage attribute slightly impacts their distribution. t-SNE. [file mmc2.pdf]

A

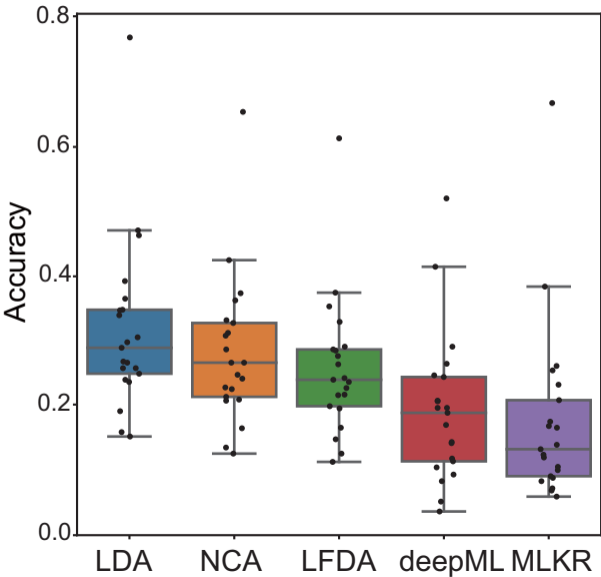

B

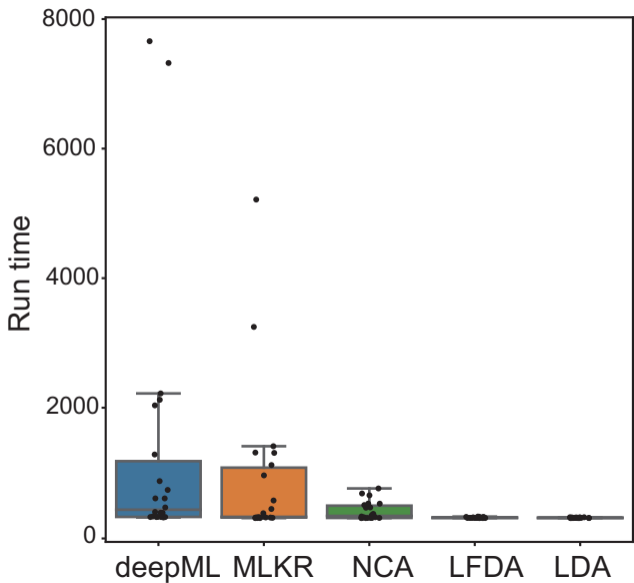

Supplement: Supplementary Figure S2 — Benchmarking the metric learning algorithm A. LDA is superior to NCA, LFDA, deepML, and MLKR in terms of accuracy in the drug annotation dataset. B. LDA is superior to NCA, LFDA, deepML, and MLKR in terms of runtime efficiency in the drug annotation dataset. LDA, linear discriminant analysis; NCA, neighborhood components analysis; LFDA, local fisher discriminant analysis; deepML, deep metric learning; MLKR, metric learning for kernel regression. [file mmc3.pdf]

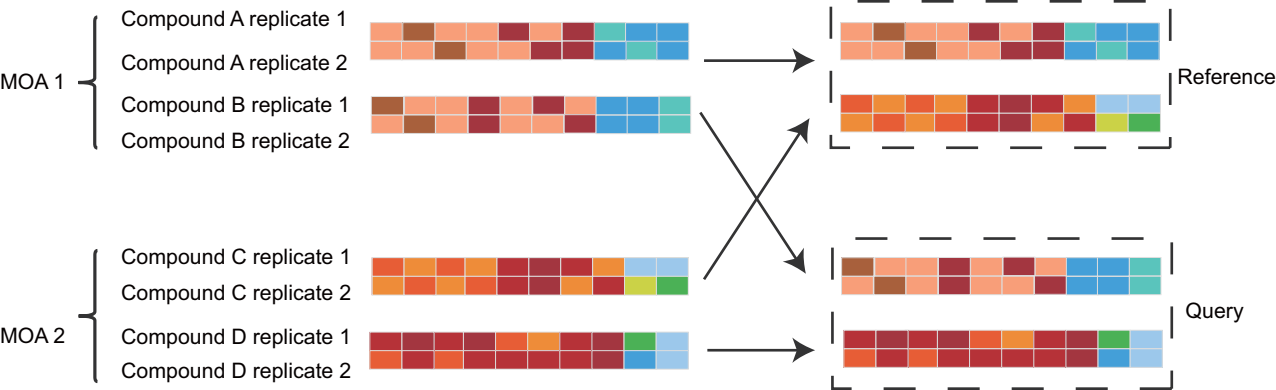

Supplement: Supplementary Figure S3 — The strategy of splitting training and testing data in the drug annotation scenario For a kind of MOA, half of the compounds were used as reference compounds and half were taken as query compounds. The signatures induced by the reference compounds were used for training and the signatures induced by the query compounds were used for testing. MOA, mechanism of action. [file mmc4.pdf]

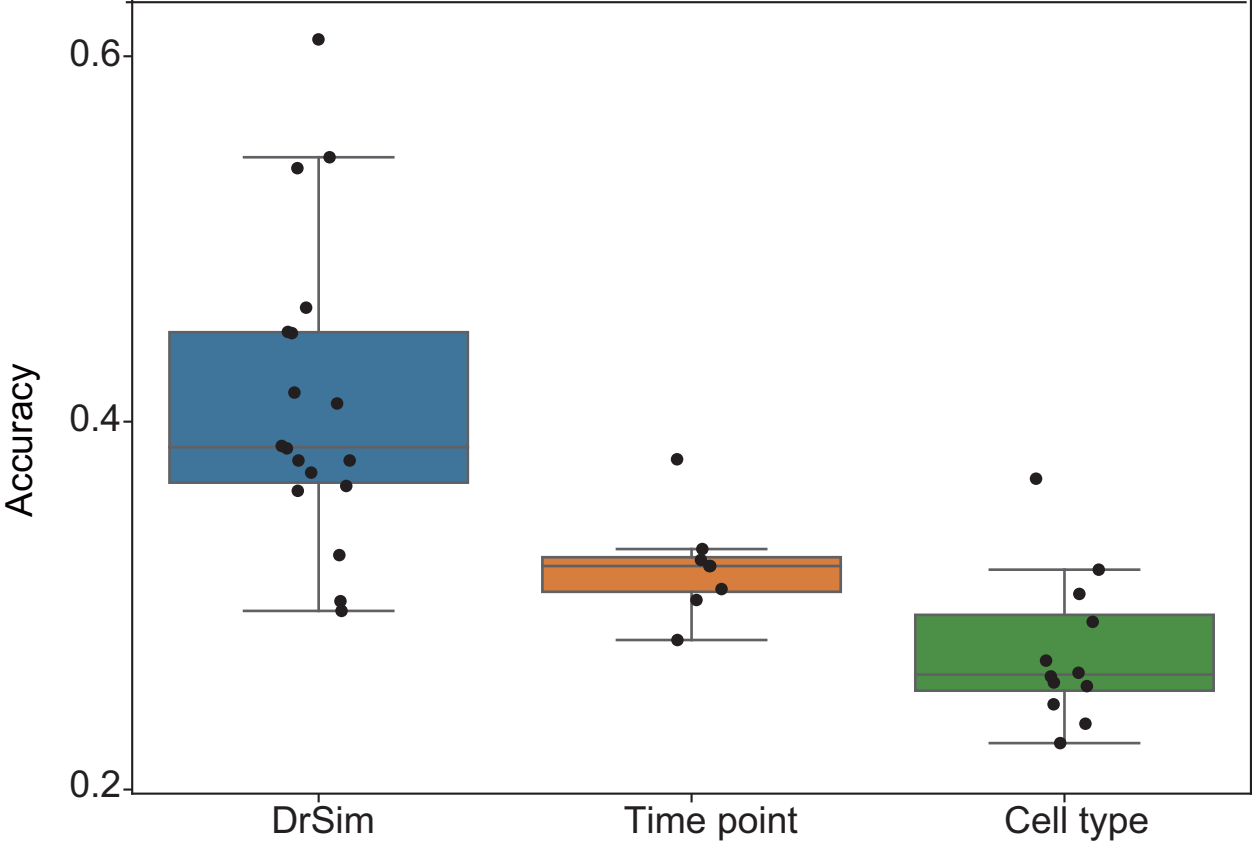

Supplement: Supplementary Figure S4 — The accuracy of predicting the MOAs of compounds if we do not take the impact of cell type and time-point attributes into consideration To evaluate the impact of cell type, signatures from different cell lines were used as queries and references, respectively. To evaluate the impact of time-point, signatures from different time points were used as queries and references, respectively. MOA, mechanism of action. [file mmc5.pdf]
